# Evidence for the Concerted Evolution between Short Linear Protein Motifs and Their Flanking Regions

**DOI:** 10.1371/journal.pone.0006052

**Published:** 2009-07-08

**Authors:** Claudia Chica, Francesca Diella, Toby J. Gibson

**Affiliations:** 1 Structural and Computational Biology Unit, EMBL Heidelberg, Heidelberg, Germany; 2 biobyte solutions GmbH, Heidelberg, Germany; Utrecht University, Netherlands

## Abstract

**Background:**

Linear motifs are short modules of protein sequences that play a crucial role in mediating and regulating many protein–protein interactions. The function of linear motifs strongly depends on the context, e.g. functional instances mainly occur inside flexible regions that are accessible for interaction. Sometimes linear motifs appear as isolated islands of conservation in multiple sequence alignments. However, they also occur in larger blocks of sequence conservation, suggesting an active role for the neighbouring amino acids.

**Results:**

The evolution of regions flanking 116 functional linear motif instances was studied. The conservation of the amino acid sequence and order/disorder tendency of those regions was related to presence/absence of the instance. For the majority of the analysed instances, the pairs of sequences conserving the linear motif were also observed to maintain a similar local structural tendency and/or to have higher local sequence conservation when compared to pairs of sequences where one is missing the linear motif. Furthermore, those instances have a higher chance to co–evolve with the neighbouring residues in comparison to the distant ones. Those findings are supported by examples where the regulation of the linear motif–mediated interaction has been shown to depend on the modifications (e.g. phosphorylation) at neighbouring positions or is thought to benefit from the binding versatility of disordered regions.

**Conclusion:**

The results suggest that flanking regions are relevant for linear motif–mediated interactions, both at the structural and sequence level. More interestingly, they indicate that the prediction of linear motif instances can be enriched with contextual information by performing a sequence analysis similar to the one presented here. This can facilitate the understanding of the role of these predicted instances in determining the protein function inside the broader context of the cellular network where they arise.

## Introduction

Linear motifs (LMs) are short stretches of amino acids that populate protein sequences and play fundamental roles in protein interaction networks [1]. Their lengths are typically between three and ten amino acids [2], [3]. LMs frequently show wide variation in residue conservation: some positions accept only one or few amino acids while others do not have any preference and function as spacers [4]. These sequence features give to LMs an evolutionary plasticity and an important role in the evolution of cellular networks by the addition of new functionality to proteins [1].

LMs are mainly found in intrinsically unstructured regions of proteins [5]. Disordered regions allow a thermodynamical control of the affinity and specificity of protein interactions. They favour transient, that is to say low affinity, and conditional interactions, often depending on a previous modification like a phosphorylation [6]. Hence the localisation of LMs in disordered regions suits dynamic regulation of protein networks, where a rapid but deterministic response is needed [7]. Indeed, LM–mediated interactions allow the emergence of several regulatory modes (i.e. sequential, mutually exclusive and cooperative) frequently observed in signalling, vesicular trafficking and transcription pathways [8].

Function of LMs strongly depends on the context. An instance of the KDEL motif, which is an endoplasmic reticulum retrieving signal, is likely to be functional only if present in protein sequences known to localise to the ER or Golgi apparatus. On one hand, the context defines the natural constraints that act on LMs and therefore provides “rules” that can be applied to evaluate the reliability of a newly predicted pattern or instance. For example the domain masking strategy, which is used to discard instances occurring in protein regions inaccessible for interaction like globular domains or coiled coils [3], [9], [10], [11].

On the other hand, the context can also give detailed information about the mode of action of LMs. The role of the local amino acid composition in determining specificity of LM interactions has been experimentally studied at the interactome level [12], [13], [14]. At the structural level, unstructured regions flanking LMs have been observed to undergo disorder to order transition upon binding [15], forming either 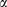 -helices [16] or additional 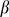 strands that join a 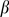 sheet of the partner [17]. This coincides with the observation that two thirds of LMs bind to their partners by mutual fit, meaning that they acquire a fixed structure upon binding to a well structured template [1]. Furthermore, a recent survey of 3D structures of protein–peptide complexes has estimated that neighbouring residues account for 20% of the global binding energy of peptide–mediated interactions. They are thought to improve the interaction affinity with the native partner or to impede non–native interactions [18].

The evolutionary context of LMs has also been studied and used in predictive methods. Convergent evolution of LMs is at the basis of discovery algorithms like SLiMFinder [19] and DILIMOT [20], which search for over–represented motifs in unrelated proteins with a common functional attribute. Additionally, conservation of LMs in closely and distantly related proteins has been used to improve the identification of functional instances of known LM patterns [11], [21], [22], [23]. Methods for *de novo* discovery, have also benefited from the evolutionary signal provided by analysing patterns of conservation. SLiMFinder uses global or local sequence conservation to improve confidence in motif predictions [9], [24]; DILIMOT takes into account conservation of the motif in orthologs as part of the scoring scheme [10].

It is clear that LM predictions from the current generation of predictors require experimental validation to be considered genuine. The methods are often working at the limits of signal to noise and are dependent on the information content of the bioinformatics databases being used for LM prediction [3], [25], [26]. Nevertheless, LM prediction methods could be valuable tools for the study of high dimensional systems like the protein signalling networks. Therefore it is necessary to move from the identification of a LM in a protein towards the prediction of the role of that instance inside the functional framework of the protein, e.g. its network of interactors.

This work addresses the study of LM context from an evolutionary point of view. Conservation patterns of regions flanking 116 LM functional instances were examined in relation to the presence/absence of the LM inside protein families. Both sequence identity and structural tendency of the LM context was analysed. Notwithstanding the difficulty of assessing the generality of the results, due to the fragmentary knowledge about the complete set of cellular LMs, distinct evolutionary patterns were identified. For the majority of the studied instances, conservation of the local amino acid sequence and/or the local structural tendency was found to be differentially distributed between sequence pairs with and without the motif. These findings are supported by examples where the regulation of the LM mediated interaction has been shown to depend on the modifications at neighbouring positions or is thought to benefit from the binding versatility of disordered regions. Taken together, the results of the present study suggest that it is possible to enrich the identification of a LM instance with regulatory information by analysing the conservation pattern of its flanking regions.

## Methods

### Dataset

The analysis was done using the MAFFT [27] alignments of 75 protein families containing 85 protein sequences that have 116 non–redundant LM instances linked to experimental evidence in the ELM database [3]. Protein families were taken from the TreeFam4.0 database [28]. The 40% of the families in the dataset include proteins of metazoans (vertebrates and invertebrates) and plants (*A. thaliana*) or yeast (*S. cerevisiae* and *S. pombe*); 42% contain vertebrate and invertebrate sequences; the remaining 18% have only vertebrate proteins.

The presence/absence of each instance was determined in the sequences belonging to the protein family by looking for the regular expression of the corresponding LM, as defined in the ELM resource [3]. Sequence pairs in the protein family were assigned to one of the following sets: the presence set (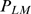), when both sequences have a match to the regular expression in the same position of the annotated ELM instance; the absence set (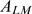), when the instance is missing in one of the sequences. Only protein sequences having a sub–sequence aligned to the region corresponding to the ELM instance were considered. This classification assumes that a LM instance is functional if it appears in a position that, according to the alignment, corresponds to that of the annotated ELM instance. Moreover, it depends on the adequacy of the ELM regular expression and might overestimate the size of the 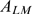 set. Sequence pairs where the instance is absent in both sequences were not considered, since any interpretation about their differences would imply making assumptions about the gain or loss of the instances during the evolution of the protein family.

To perform comparisons between LMs located in similar structural contexts, each instance was assigned to a structural class. The structural class was defined in terms of disorder/order at two levels: protein family and module, where module is defined as an independent unit within the protein sequence with globular or disorder tendency. This classification was done in a semi–automated way, using the IUPred disorder predictor [29] and the SMART module research tool [30] and averaging the results over all the homologous sequences. Proteins were classified as disordered, when more than 70% of their residues are disordered (conservative IUPred threshold of 0.4); globular, when more than 70% of the residues belong to one or more SMART globular modules; mixed, for the proteins that could not be clearly allocated to any of the previous classes. Modules were similarly defined as disordered or globular. The final dataset has instances in all of the 6 structural classes resulting from the combination of protein and module class (see Text S1 for the complete dataset).

### Local structure and sequence conservation metrics

Differences between sequences were studied in terms of conservation of the local structural tendency and the amino acid sequence at both local and global level. The conservation of the local structure was calculated for each sequence pair 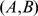 as:
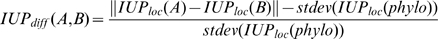
where 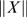 indicates the absolute value of 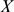; 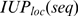 is the IUPred value averaged over the amino acids located 15 positions to the left and right of the LM in sequence 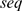; 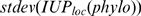 is the standard deviation of 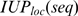 for all the sequences in the protein family. Therefore, 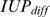 indicates whether the difference of the local tendency to disorder/order between A and B is higher or lower than the variability inside the whole protein family. Normalisation by standard deviation permits the comparison among instances belonging to different protein families, which have different IUPred variabilities. The 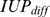 varies between −1 and infinity, with negative or small positive values indicating conservation of the local structural tendency around the LM instance.

The protein sequence conservation between each pair 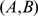 was calculated as the full-length sequence identity according to the multiple sequence alignment (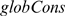) and as the sequence identity of the amino acids in the 15 positions flanking the LM instance both sides (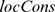).

The definition of 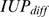 and 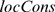 depends on the alignment quality of the flanking regions. Acknowledging the poor performance of multiple alignment programs in disordered regions [31], those values were calculated only when the 15 residue windows surrounding the instance contained at least 75% of non–gap positions; in other words, when there was enough information to estimate average conservation values.

### Frequency profiles and correlation between 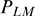 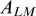 sets

The distribution of the 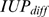 values as a function of the 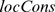 or 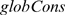 was represented as frequency profiles. Those profiles are no more than two-dimensional histograms which represent the number of pairs falling in a given range of the 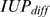 and a given range of 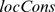 or 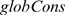. Counts were normalised to avoid biases due to the different sizes of the protein families. Frequency profiles were calculated for the 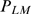 and 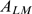 sets of each instance. Almost half of the instances (53 out of the 116) have a sufficient number of sequence pairs to allow this statistical representation.

In order to compare the similarity between the 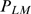 and 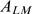 profiles, their correlation was estimated using the Spearman coefficient. The Spearman coefficient ranges between 1, high correlation, and −1 complete anticorrelation. In the context of the present study, a correlation of 1 would indicate that the 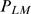 and 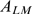 sets cover the same 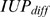 and 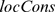/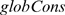 ranges. A correlation of −1 would imply that those ranges are completely disjoint and diametrically opposed (e.g. high 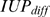 and low 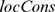 for 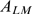 while low 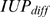 and high 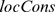 for 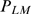). Small positive or negative values indicate that the 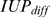 and 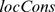/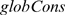 ranges of the 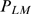 and 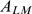 sets tend to be disjoint but not opposite.

### Statistical coupling analysis

Positional coupling [32] between each non–wildcard position of the LM instance and each one of the residues of the module (globular or disordered) was calculated. The method could be applied for the instances located in modules whose multiple sequence alignment is diverse, such that the frequencies of amino acids at some positions are near to their mean values in all proteins, i.e. those positions are poorly conserved. Only positions in the module with coupling values that emerge from noise were considered. Noise threshold was set to two standard deviations above the mean coupling value of all the residues in the module.

Coupled positions were classified as neighbouring, when located within 15 positions both sides of the LM instance, and as distant for all the others. For the instances located towards the limits of the module, the partial window (i.e. less than 15 residues) was considered. In other words, the module boundaries were taken into account when defining neighbouring residues.

Assuming that the probability of coupling is equal for any residue in the protein sequence, the number of coupled positions was weighted by the total number of potentially coupled positions: 30 for the neighbouring residues and the length of the module minus the length of the instance region (15+ motif length +15) for the distant ones. This weighted value is defined as the frequency of coupling.

## Results

### LM presence and the conservation of the local structural tendency

**Figure 1:**
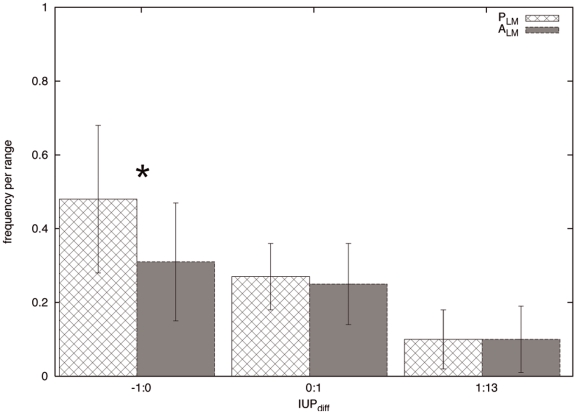
Frequency distribution of *IU P_diff_* for the *P_LM_* and *A_LM_* sets. Frequency is calculated per instance as the proportion of sequence pairs falling in a given *IU P_diff_* range. Error bars indicate the standard deviation of the frequency when averaging over all the instances in that range. Significant difference (p-value<0.00001) between *P_LM_* and *A_LM_* distributions is marked by the asterisk.

This section explores the relationship between LM presence and the conservation of the structural tendency in the regions flanking the motif. Figure 1 shows the 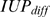 distribution for the pairs of the 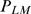 and the 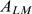 sets averaged over all the instances. Even if there is a non–negligible overlap between the two distributions, negative 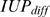 values, that indicate conservation of the local structural tendency, are significantly more frequent in 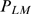 than in 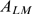 sequence pairs (Kolmogorov-Smirnov test: difference = 0.423, p-value
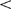
0.00001). This difference is lost for higher 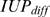 values.

When the analysis is repeated comparing the 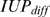 distributions of 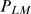 and 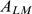 sets of each instance, inside each protein family, analogous results are obtained. For all the structural classes the mean 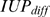 for the 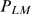 set is lower than that of the 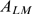 set, as shown in Table 1. Additionally, comparison of the two 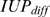 distributions gives statistically significant differences for 57 out of 116 instances (Kolmogorov-Smirnov test: differences between 0.303 and 0.791, p-values
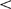
0.05, see complete results in Table S1). This means that, for almost 50% of the instances the 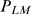 and 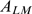 sets have different local structural tendencies that can be quantified and used to statistically differentiate between those sequence pair sets.

**Table 1:** *IU P_diff_* ranges and mean *IU P_diff_* for the *P_LM_* and *A_LM_* sets per structural class.

protein class	module class	number^a^	min		max		mean	
			*P_lm_*	*A_lm_*	*P_lm_*	*A_lm_*	*P_lm_*	*A_lm_*
DIS	DIS GLOB	41	−0.9	−0.8	3.4	4.9	0.6	1.2
		4	−1.0	−1.0	1.8	3.5	0.0	0.6
GLOB	DIS GLOB	16	−0.9	−0.9	3.9	6.9	0.6	1.6
		14	−1.0	−0.8	2.1	5.2	0.1	1.2
MIXED	DIS GLOB	32	−0.9	−0.9	3.2	6.0	0.5	1.8
		9	−1.0	−0.9	2.1	4.6	0.2	1.3

*IU P_diff_* values are averaged over all the instances belonging to the same structural class.

a number of instances per structural class.

For the remaining instances the 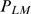 and 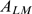 sets have the same 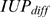 ranges. These instances suggest that, sometimes, the local structure is conserved even if the LM is lost. This is not surprising if considering that the LM is a module evolving inside a higher order unit (e.g. the protein sequence) composed of several other functional modules. Disambiguation of the selective pressure imposed by the LM, based exclusively in its local structure conservation, will be difficult in these cases. Consequently it is worth analysing the conservation of the local structural tendency in relation to the evolution of the rest of the protein modules.

### LM evolution and the relationship between local structural tendency and sequence conservation

In order to explore how the conservation of the local structure, in terms of disorder/order, is related to the evolution of the protein sequence, the distribution of 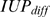 was analysed as a function of the global and local sequence conservation. Frequency profiles of the combined distribution of 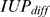 versus the local and global sequence conservation (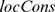 and 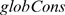) were calculated for both the 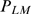 and 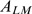 sets of each instance.

**Figure 2:**
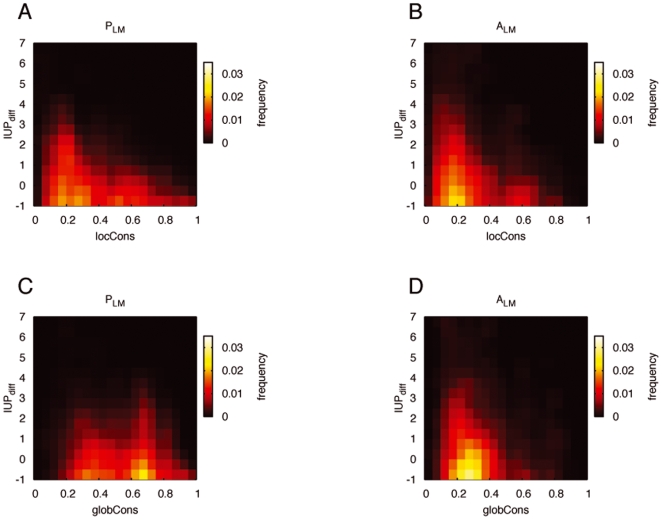
Frequency profiles for the *P_LM_* and *A_LM_* sets. Distribution of *IU P_diff_* as a function of sequence conservation: *locCons* (A,B) and *globCons* (C,D). Colour represents the frequency of sequence pairs whose local structure and sequence conservation values fall in a given range of *IU P_diff_* and *locCons/globCons*, averaged over all the instances.

Figure 2 presents the frequency profile of 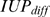 versus 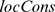 and 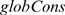. Since they represent the distribution of the above variables for the 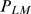 and 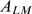 sets averaged over all the instances, those profiles do not allow a comparative analysis between 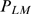 and 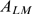 sets or sequence conservation variables. Differences among protein families due to dissimilar evolutionary rates are not averaged out. The structural composition of proteins belonging to different structural classes (disordered, globular, mixed) might add further disparity, since sequences with long disordered regions tend to have heterogeneous evolutionary rates [33].

Nevertheless those profiles provide an idea about the general trends of the relationship between 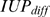 and sequence conservation. As expected, the 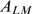 sets cover mainly low sequence conservation values (Figure 2B and D). Indeed, even if low sequence similarity does not necessarily imply the loss of the LM, closely related protein sequences are more likely to have similar LM instances than distantly related or paralogous sequences [1], [4]. Instead, the frequency profiles of the 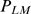 sets exhibit an additional feature: low 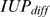 values are frequent in both high and low sequence conservation values (Figure 2A and C). In other words, conservation of the amino acid sequence is not required for the maintenance of the disorder tendency around the LM.

The above result suggests that structural and sequence conservation, intended as sequence identity, are not redundant and both might provide information about the LM evolution. Indeed the IUPred method predicts disordered/ordered regions by estimating the total pair wise interresidue interaction energy [29] and therefore there is no *a priori* reason why the conservation of the local structural tendency should imply the conservation of the exact amino acid sequence. To further explore this, the frequency profiles of the 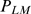 and 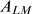 sets of each instance were obtained and their Spearman correlation coefficient calculated separately. The analysis per instance has the additional advantage of discarding artificial differences between 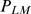 and 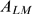 caused by dissimilar evolutionary rates among the protein families.

**Figure 3:**
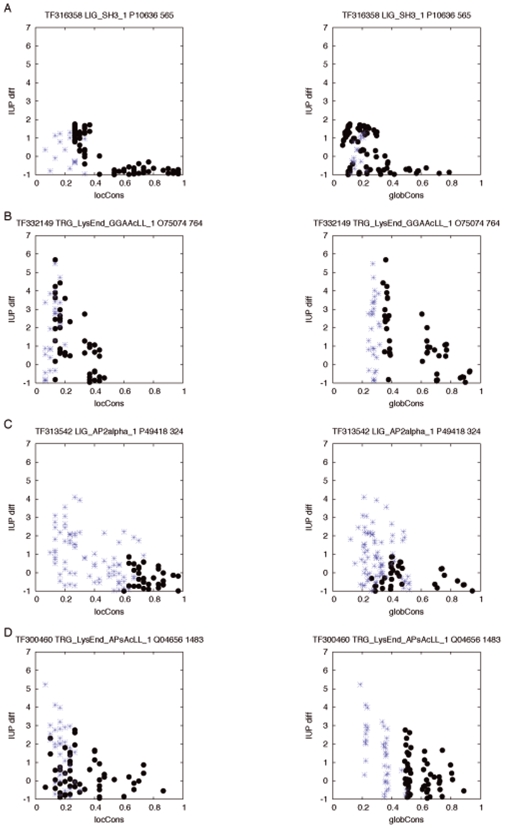
Examples of evolutionary patterns of the regions flanking LM. *IU P_diff_* versus *locCons* and *globCons* for the sequence pairs in *P_LM_* (black dots) and *A_LM_* (blue asterisks) sets per instance. Three groups with distinct evolutionary behaviour can be identified: instances whose *P_LM_* and *A_LM_* frequency profiles of *IU P_diff_* versus *locCons* are less correlated than the corresponding *IU P_diff_* versus *globCons* profiles (A); instances where the contrary is true (B); instances that, additionally, have a significantly different *IU P_diff_* distribution (C,D).

All the structural classes have low mean correlation coefficients indicating that, on average, the 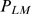 and 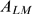 frequency profiles of each instance can be discriminated; correlation values range from 0.11 to 0.34 for 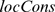 and from 0.02 to 0.22 for 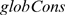 depending on the structural class (see Table S2). The low number of instances per structural class, makes any comparative statistical analysis unreliable, e.g. between structural classes or conservation variables. Nevertheless, having a closer look at the results per instance (Table 2), three groups with distinct behaviour can be identified. Examples of instances belonging to each one of those groups are presented in Figure 3. Those trends do not change when the 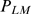 set is enlarged by considering subsequences that partially match the ELM regular expression as LM instances (see Table S3 for further details).

**Table 2:** Spearman correlation coefficient between the *P_LM_* and *A_LM_* frequency profiles.

structural class^a^	TreeFam id	UniProt id	ELM id	Start	*locCons* corr	*globCons* corr
	TF106427	P29374	LIG_RB	957	−0.14	0.12
	TF106496	P25054	TRG_NES_CRM1_1	163	−0.09	−0.05
	TF316358	P10636	LIG_SH3_1	565	−0.05	0.42
	**TF300785**	**P51531**	**LIG**_**RB**	**1294**	**−0.01**	**0.12**
	**TF314303**	**O15147**	**LIG**_**SH3**_**5**	**389**	**−0.01**	**0.16**
	**TF325994**	**P35568**	**LIG**_**14-3-3**_**3**	**267**	**0.12**	**0.29**
	TF331759	O60315	LIG_CtBP	785	0.16	0.20
	TF323952	P17535	LIG_COP1	241	0.16	0.33
DIS DIS	TF325994	P35568	LIG_14-3-3_3	371	0.29	0.42
	TF318445	O35973	TRG_NES_CRM1_1	488	0.32	0.50
	TF325994	P35570	LIG_SH2_GRB2	896	0.45	0.52
	TF101166	P05205	LIG_RB	61	0.53	0.10
	**TF320471**	**P35712**	**LIG**_**CtBP**	**424**	**0.36**	**0.15**
	TF313876	Q91VZ6	LIG_Clathr_ClatBox_l	192	0.31	0.16
	TF325994	P35570	LIG_SH2_PTP2	1179	0.23	0.21
	TF331759	O60315	LIG_CtBP	859	0.34	0.31
	TF323952	P05412	MOD_PIKK_l	246	0.55	0.52
	**TF105306**	**Q00987**	**MOD**_**PIKK**_**l**	**392**	**−0.02**	**0.02**
DIS GLOB	TF323952	P05412	LIG_MAPK_1	32	0.55	0.27
	**TF314861**	**Q05140**	**LIG_PIP2_ANTH_1**	**28**	**0.51**	**0.36**
	TF325994	P35570	MOD_CK2_1	96	0.48	0.39
	**TF335892**	**P04235**	**TRG**_**LysEnd**_**APsAcLL**_**l**	**138**	**0.18**	**0.28**
	**TF300460**	**Q04656**	**TRG_LysEnd_APsAcLL**_**l**	**1483**	**0.38**	**0.00**
	TF105137	Q02750	LIG_MAPK_1	3	0.34	0.11
	TF300618	P27797	TRG_ER_KDEL_l	414	0.53	0.22
GLOB DIS	TF105135	P45985	LIG_MAPK_1	40	0.36	0.28
	**TF105115**	**Q99683**	**LIG**_**14-3-3**_**1**	**963**	**0.33**	**0.31**
	**TF300540**	**P04040**	**TRG**_**PTS1**	**523**	**0.38**	**0.36**
	**TF105044**	**P36604**	**TRG_ER_KDEL_1**	**660**	**0.45**	**0.42**
	TF106381	P09103	TRG_ER_KDEL_l	506	0.52	0.48
	TF105042	P17156	LIG_TPR	630	0.65	0.52
	**TF335892**	**P19377**	**MOD_TYR_ITAM**	**146**	**−0.05**	**−0.08**
	TF101211	Q8AY27	MOD_PIKK_l	2	0.03	0.00
	**TF101004**	**P24385**	**LIG**_**RB**	**5**	**0.22**	**0.10**
GLOB GLOB	**TF105115**	**Q99683**	**LIG**_**RB**	**916**	**0.19**	**0.17**
	**TF105122**	**P28562**	**LIG**_**MAPK**_**2**	**339**	**0.44**	**0.25**
	TF315491	P27918	MOD_CMANNOS	318	0.37	0.27
	TF105331	Q96GD4	LIG_APCC_Dbox_l	314	0.65	0.53
	**TF316520**	**O00268**	**LIG**_**HP1**_**1**	**762**	**−0.07**	**0.01**
	**TF101065**	**Q12834**	**LIG_APCC_KENbox_2**	**96**	**−0.01**	**0.04**
	**TF313542**	**P49418**	**LIG**_**AP2alpha_l**	**324**	**0.03**	**0.15**
	TF300772	P49736	MOD_PIKK_1	105	0.09	0.16
MIXED DIS	**TF105351**	**P35465**	**LIG**_**SH3**_**2**	**13**	**0.11**	**−0.06**
	TF332149	O75074	TRG_LysEnd_GGAAcLL_l	764	0.35	0.01
	**TF106101**	**P04637**	**TRG_NES_CRM1_1**	**339**	**0.04**	**0.01**
	TF318574	Q9UJY5	TRG_LysEnd_GGAAcLL_2	355	0.34	0.03
	**TF101089**	**P53350**	**LIG**_**APCC**_**Dbox**_**l**	**336**	**0.33**	**0.14**
	TF105722	P35251	LIG_RB	662	−0.11	−0.06
	**TF300901**	**P23396**	**LIG**_**MAPK**_**2**	**77**	**0.24**	**0.41**
MIXED GLOB	**TF333209**	**P54274**	MOD_PIKK_1	**216**	**−0.08**	**−0.10**
	**TF318283**	**P46061**	**MOD**_**SUMO**	**525**	**0.10**	**−0.07**
	**TF101066**	**Q8UWJ8**	**LIG**_**CYCLIN**_**l**	**445**	**0.31**	**0.19**
	**TF330851**	**P10912**	**LIG**_**SH2**_**STATB**	**566**	**0.30**	**0.21**

Spearman correlation coefficient calculated between the *P_LM_* and *A_LM_* frequency profiles of each instance. Correlation of the frequency profiles of *IU P_diff_* versus *locCons* and *IU P_diff_* versus *globCons* are indicated as *locCons* corr and *globCons* corr respectively. Correlation of 1 would indicate that the *P_LM_* and *A_LM_* sets cover the same *IU P_diff_* and *locCons/globCons* ranges. A correlation of −1 would imply that those ranges are completely disjoint and diametrically opposed (e.g. high *IU P_diff_* and low *locCons* for *A_LM_* while low *IU P_diff_* and high *locCons* for *P_LM_*). Small positive or negative values indicate that the ranges tend to be disjoint but not opposite. Instances in bold have *P_LM_* and *A_LM_* sets with significantly different *IU P_diff_* distributions (p-values<0.05).

a protein and module structural classes.

The first group consists of those instances whose 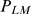 and 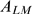 frequency profiles of 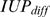 versus 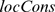 are less correlated than the corresponding 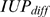 versus 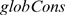 profiles (Figure 3A). This indicates that variations in the local protein sequence are more connected to the LM presence/absence than the modifications happening in the rest of the protein. The 37% of the instances in Table 2 have this kind of behaviour, especially those ones located in disordered modules of disordered proteins (8 out of 13).

The second group is formed of instances where the contrary is true, meaning that the LM presence/absence is better distinguished by the global conservation (Figure 3B). In those cases, the main selective pressure on the LM presence might be coming from the protein sequence as a whole unit. Not surprisingly all of the 8 instances located in globular proteins (both in disordered and globular modules) belong to this group.

A third group of instances appears when merging the results of the previous section, that is to say, considering those instances whose 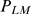 and 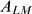 sets have significantly different 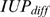 distributions (in bold in Table 2, Figure 3C and D). In these cases, the presence or absence of the LM is correlated with changes in both the local structural tendency **and** the sequence conservation. Those instances reach, on average, lower correlation values independently from the conservation variable (0.18 for the 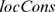 and 0.15 for the 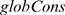) than the instances with no significant 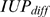 distinction between 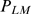 and 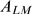 (0.30 for 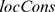 and 0.26 for 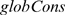). This last group of instances is the best evidence in favour of the hypothesis proposed above, about the additive value of the structural and sequence conservation information in the analysis of LM evolution.

### Co-evolution of the LM and their flanking regions

To get additional evidence about the co–evolution between LMs and their flanking regions, the statistical coupling [32] was used as an independent method. This method has been used to identify clusters of positions that statistically co–vary with one another and therefore are likely to co–evolve and to be functionally related [34]. In this case only pair coupling between the non–wildcard positions of the LM instance and all the other residues in the corresponding module was considered. The frequency of coupling with neighbouring and distant residues was calculated and compared in terms of the sequence conservation that best describes the LM evolution, that is to say the variable that gives the lowest correlation in Table 2.

**Figure 4:**
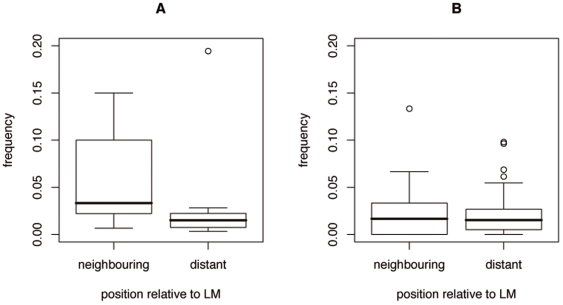
Frequency of coupling between LM and neighbouring or distant residues. Box plots show the distribution of the frequency of coupling for instances in Table 2. A. Distribution for instances whose presence/absence is better described by the local rather than the global sequence conservation (i.e. *locCons* correlation<*globCons* correlation) B. Distribution for instances with *globCons* correlation<*locCons* correlation.

For the instances that have lower 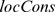 correlation (e.g. Figure 3A), the frequency of neighbouring coupling is significantly higher (Kolmogorov-Smirnov test: difference = 0.576, p-value
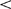
0.005) than the frequency of distant coupling (Figure 4A). In other words, the instances whose evolution is better described by the local sequence conservation combined with the 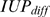 have a higher chance of correlated amino acid changes with neighbouring rather than with distant residues in the module. Conversely, for the instances where the global sequence conservation is the better descriptor (e.g. Figure 3B), the coupling between non–wildcard positions and neighbouring or distant positions is equally frequent (Figure 4B).

## Discussion

This study presents evidence for the concerted evolution of LMs and their flanking regions. Although the current knowledge of the complete set of cellular LMs is fragmentary and it is not possible to assess the representativity of the analysed dataset, there are clear trends that are worth considering. LMs are known to be evolutionarily labile modules, which can be easily lost by point mutation [4]. Nonetheless, the results of the present study show that LMs, in some cases, determine the conservation of the structural tendency and/or the sequence of the neighbouring amino acids. Here those findings are discussed in the light of the protein interactions mediated by LMs.

In the first section of the Results it was shown that, for some instances, the conservation of the LM is associated with the maintenance of the structural tendency of the surrounding residues. What is the meaning of this conservation? As mentioned in the Introduction, two thirds of the LM–mediated interactions lead to the formation of secondary structure elements (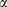
–helices or 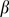
–strands) [1]. If the LM functionality is to be maintained, the structural properties of the neighbouring amino acids that allow such disorder/order transition are likely to be conserved. This local propensity would be reflected by the corresponding IUPred values and hence the low 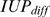 observed in the 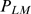 sets would indicate the conservation of such propensity.

However, the conservation of the local structural tendency could also indicate the maintenance of the local disorder. Several studies on protein–protein interactions have drawn attention to the importance of intrinsic disorder in the formation of protein complexes [6], [35], [36], [37]. If the local disorder provides the flexibility required to bind different patterns, it is not surprising to observe the conservation of this structural tendency in the regions involved in such interactions. Previous work by [38] has connected the conservation of predicted disordered regions in eukaryotic proteins with DNA/RNA binding domains. The conservation of disorder around LMs would extend this result to a broader set of biological processes.

The instances of the molecular hub p53 exemplify the double meaning of the structural conservation measured by the *IU P_diff_*. For three out of four of the p53 instances in the dataset (TRG_NES_CRM1_1, 339–352; MOD_SUMO, 385–388; MOD_PIKK_1, 12–18), the presence of the instance coincides with the conservation of the local structural tendency. They belong to the group of instances that have a significantly different distribution of the 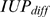 between 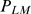 and 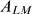 sets (p-value
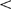
0.05). Those instances are located in the C and N terminal regions of P53, which are disordered modules known to bind different partners by acquiring different conformations [39]. Additionally, the MOD_SUMO and the MOD_PIKK_1 (but not the TRG_NES_CRM1_1) occur in predicted *α*–MoREs, disordered regions having propensities to form *α*–helix upon molecular recognition [16].

A more detailed study of the structural conservation as function of the different types of mutual fit interaction (i.e. *α*–helix formation, 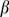 augmentation or irregular topology) may be interesting. It would shade light on the specific requirements of each conformation. This would require the definition of a more elaborated metric for the local structure conservation than the 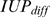. However, independently from its specific meaning, the structural tendency conservation around the LM suggests the occurrence of overlapping interaction surfaces. Those clustered overlaps are likely to entail different regulatory mechanisms for the spatial or temporal isolation of the mutually exclusive interactions.

In the second and third part of the Results it was shown that the presence of some LM instances is accompanied by the conservation of the amino acids flanking the motif. This is the case for 42% of the instances in Table 2 that have 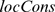 correlation values lower that 0.20 between the 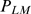 and 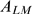 sets. The local sequence conservation could be explained in some cases by the conservation of the local structural tendency (instances in bold in Table 2, Figure 3C and D). Still, as shown in the Results (Figure 2), sequence identity does not seem to be a requirement for the maintenance of the local order/disorder tendency. Indeed, it has been recently demonstrated by nuclear magnetic resonance spectroscopy that intrinsically disordered regions can maintain their dynamic behaviour despite low sequence similarity [40]. Yet there must be a functional meaning for the local sequence conservation associated with these instances, especially considering that it allows to discriminate sequences with and without the motif (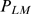 and 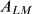 sets), even when local structural tendencies between those sequences are not significantly different (e.g. Figure 3A and B). Furthermore, these instances have higher chance of co–evolving with the neighbouring residues in comparison to the distant ones (Figure 4A).

It is likely that the flanking regions of those instances are related with the regulation of the LM or with the regulation of another interaction, which is functionally connected to the one mediated by the motif. This is the case of the LIG_AP2alpha_1 in positions 324–328 of amphiphysin (P49418, 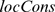 correlation 0.03), which is involved in clathrin coated vesicle formation. Phosphorylation of amphiphysin by Cdk5 in S276, S285 and T310 has been shown to directly regulate the intramolecular interaction in amphiphysin, which in turn regulates dynamin-dependent endocytosis [41], [42]. Likewise, other instances with 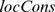 correlation between −0.05 and 0.16 (LIG_SH3_1 P10636 565–572, LIG_COP1 P17535 241–248) have experimentally verified phosphorylation sites in their flanking regions: T561 for P17535 and S251, S255 and S259 for P17535 [25]. Those phosphorylation site are likely to regulate the local protein conformation and activity, as recently shown in a phosphoproteomic analysis of the mouse brain cytosol [43].

Finally, it is opportune to consider how current LM prediction methods can benefit from these results. A simple sequence analysis, similar to the one described here, would allow the identification of flanking regions with relevant conservation patterns, adding contextual information to already predicted LM instances. This can lead to a more detailed understanding of the role of LMs in determining the protein function. Indeed we consider that the LM field is ready – and has the potential – to go one step further from the timeless binary interactions towards the construction of more dynamic and realistic protein networks.

## Supporting Information

Text S1 Dataset of functional instances. List of the 116 instances, classified per structural class with phylogeny, sequence and motif identifiers.
(0.00 MB TXT)

Table S1 Comparison of the *IUP_diff_* distribution between the *P_LM_* and *A_LM_* sets. Kolmogorov-Smirnov test comparing the *IUP_diff_* distribution of the *P_LM_* and *A_LM_* sets of each instance. The difference is the Kolmogorov-Smirnov statistic calculated from the cumulative distributions of the compared samples.
(0.03 MB PDF)

Table S2 Mean and standard deviation of the correlation between *P_LM_* and *A_LM_* frequency profiles. Spearman correlation coefficient calculated between the *P_LM_* and *A_LM_* frequency profiles of each instance. Correlation of the frequency profiles of *IUP_diff_* versus *locCons* and *IUP_diff_* versus *globCons* are indicated as *locCons* corr and *globCons* corr respectively.
(0.02 MB PDF)

Table S3 Effect of the stringency of the regular expression matching on the correlation between the *P_LM_* and *A_LM_* frequency profiles. Spearman correlation coefficient calculated between the *P_LM_* and *A_LM_* frequency profiles of each instance. Correlation of the frequency profiles of *IUP_diff_* versus *locCons* and *IUP_diff_* versus *globCons* are indicated as *locCons* corr and *globCons* corr respectively. Percentages indicate the stringency used to define a match to the ELM regular expression: 100% stringency supposes that a LM is present only if there is a perfect match to the ELM regular expression in the same position of the annotated instance; lower percentages consider that a LM is present also in case of partial match to the regular expression. Correlation values in bold show the biggest difference (more than 0.05) with the corresponding 100% stringency correlation value. Missing values can not be calculated due insufficient number of sequence pairs in the *A_LM_* set.
(0.05 MB PDF)
