# Supplementary material for: Evidence for the Concerted Evolution between Short Linear Protein Motifs and Their Flanking Regions
Source: PLoS One. 2009 Jul 8;4(7):e6052. doi: 10.1371/journal.pone.0006052 (PMC2702822; doi:10.1371/journal.pone.0006052)
Supplement: Table S1 — Comparison of the IUPdiff distribution between the PLM and ALM sets. Kolmogorov-Smirnov test comparing the IUPdiff distribution of the PLM and ALM sets of each instance. The difference is the Kolmogorov-Smirnov statistic calculated from the cumulative distributions of the compared samples. (0.03 MB PDF) [file pone.0006052.s002.pdf]

Table S1. Comparison of the IUP_{diff} distribution between the P_{LM} and A_{LM} sets

structural class[a]	TreeFam id	UniProt id	ELM id	start	difference	p-value
DIS DIS	TF314303	O15147	LIG_SH3_5	389	0.632	0.000
	TF325994	P35568	LIG_14-3-3_3	267	0.478	0.000
	TF106174	P56524	LIG_14-3-3_3	629	0.407	0.000
	TF324293	P42566	LIG_AP2alpha_2	692	0.485	0.001
	TF324293	P42567	LIG_AP2alpha_2	629	0.491	0.001
	TF314861	Q05140	LIG_AP2alpha_2	400	0.667	0.001
	TF320471	P35712	LIG_CtBP	424	0.415	0.002
	TF315309	Q03112	LIG_CtBP	584	0.517	0.002
	TF324293	P42566	LIG_AP2alpha_2	709	0.467	0.002
	TF106461	P02836	LIG_EH1	174	0.478	0.004
	TF324293	P42566	LIG_AP2alpha_2	672	0.427	0.005
	TF106174	Q99N13	LIG_CtBP	19	0.456	0.005
	TF319589	Q96JN0	LIG_CtBP	173	0.573	0.006
	TF318482	P11831	MOD_PIKK_1	432	0.609	0.006
	TF300785	P51531	LIG_RB	1294	0.229	0.006
	TF324293	P42566	LIG_AP2alpha_2	737	0.490	0.011
	TF101235	P07276	LIG_PCNA	995	0.487	0.019
	TF106496	P25054	TRG_NES_CRM1_1	163	0.533	0.050
	TF323952	P17535	LIG_COP1	241	0.201	0.051
	TF314861	Q05140	LIG_AP2alpha_2	474	0.318	0.061
	TF313876	Q91VZ6	LIG_Clathr_ClatBox_1	192	0.184	0.084
	TF106496	P25054	TRG_NES_CRM1_1	67	0.362	0.159
	TF316358	P10636	LIG_SH3_1	565	0.280	0.203
	TF106427	P29374	LIG_RB	957	0.126	0.287
	TF331342	Q8IX07	LIG_CtBP	792	0.297	0.317
	TF325994	P35570	LIG_SH2_GRB2	896	0.130	0.386
	TF330809	P61925	TRG_NES_CRM1_1	36	0.230	0.391
	TF325994	P35568	LIG_14-3-3_3	638	0.178	0.407
	TF323952	P05412	MOD_PIKK_1	246	0.125	0.412
	TF331759	O60315	LIG_CtBP	859	0.212	0.415
	TF318445	O35973	TRG_NES_CRM1_1	488	0.129	0.434
	TF334173	P16070	TRG_LysEnd_APsAcLL_1	708	0.280	0.564
	TF331660	Q9CW46	LIG_RRM_PRI_1	400	0.190	0.659
	TF101103	O60934	MOD_PIKK_1	340	0.246	0.721
	TF101166	P05205	LIG_RB	61	0.062	0.833
	TF331759	O60315	LIG_CtBP	785	0.106	0.841
	TF325994	P35570	LIG_SH2_PTP2	1179	0.095	0.870
	TF106174	Q9UQL6	LIG_CtBP	57	0.140	0.921
	TF101171	O93355	LIG_APCC_Dbox_1	32	0.161	0.939
	TF106174	Q8C2B3	LIG_CtBP	16	0.182	0.949
	TF325994	P35568	LIG_14-3-3_3	371	0.062	0.960
DIS GLOB	TF105306	Q00987	MOD_PIKK_1	392	0.717	0.000
	TF314861	Q05140	LIG_PIP2_ANTH_1	28	0.396	0.002
	TF323952	P05412	LIG_MAPK_1	32	0.125	0.466
	TF325994	P35570	MOD_CK2_1	96	0.118	0.870
GLOB DIS	TF105115	Q99683	LIG_14-3-3_1	963	0.557	0.000
	TF335892	P04235	TRG_LysEnd_APsAcLL_1	138	0.781	0.000
	TF324918	Q99523	TRG_LysEnd_GGAAcLL_1	826	0.750	0.000
	TF105044	P36604	TRG_ER_KDEL_1	660	0.401	0.000
	TF300460	Q04656	TRG_LysEnd_APsAcLL_1	1483	0.409	0.001
	TF102033	P27986	LIG_SH3_2	308	0.386	0.002
	TF300540	P04040	TRG_PTS1	523	0.234	0.041
	TF105137	P06784	LIG_MAPK_1	9	0.225	0.058
	TF300416	O15118	TRG_LysEnd_APsAcLL_1	1271	0.413	0.089
	TF106381	P09103	TRG_ER_KDEL_1	506	0.127	0.154
	TF101051	Q99741	LIG_CYCLIN_1	94	0.433	0.173
	TF300618	P27797	TRG_ER_KDEL_1	414	0.137	0.288
	TF105137	Q02750	LIG_MAPK_1	3	0.154	0.319
	TF105135	P45985	LIG_MAPK_1	40	0.115	0.338
	TF105042	P17156	LIG_TPR	630	0.055	0.444
	TF102033	P27986	LIG_SH3_1	88	0.171	0.608

GLOB GLOB	TF335892	P19377	MOD_TYR_ITAM	146	0.929	0.000
	TF105115	Q99683	LIG_RB	916	0.406	0.000
	TF105122	P28562	LIG_MAPK_2	339	0.383	0.000
	TF315491	P27918	MOD_CMANNOS	199	0.554	0.002
	TF101004	P24385	LIG_RB	5	0.319	0.003
	TF315491	P27918	MOD_CMANNOS	385	0.542	0.005
	TF315491	P27918	MOD_CMANNOS	382	0.524	0.008
	TF315491	P27918	MOD_CMANNOS	83	0.545	0.019
	TF315491	P27918	MOD_CMANNOS	318	0.346	0.235
	TF315491	P27918	MOD_CMANNOS	196	0.333	0.291
	TF105331	Q96GD4	LIG_APCC_Dbox_1	314	0.065	0.535
	TF101211	Q8AY27	MOD_PIKK_1	2	0.202	0.651
	TF315491	P27918	MOD_CMANNOS	321	0.214	0.671
	TF315491	P27918	MOD_CMANNOS	139	0.210	0.828
MIXED DIS	TF105351	P35465	LIG_SH3_2	13	0.717	0.000
	TF105165	Q27974	LIG_AP2alpha_2	674	0.780	0.000
	TF105165	Q27974	LIG_AP2alpha_2	579	0.729	0.000
	TF105165	Q27974	LIG_AP2alpha_2	605	0.791	0.000
	TF101065	Q12834	LIG_APCC_KENbox_2	96	0.602	0.000
	TF313542	P49418	LIG_Clathr_ClatBox_1	351	0.722	0.000
	TF313542	P49418	LIG_AP2alpha_1	324	0.471	0.000
	TF105568	P06400	LIG_CYCLIN_1	873	0.754	0.000
	TF316520	O00268	LIG_HP1_1	762	0.744	0.000
	TF101089	P53350	LIG_APCC_Dbox_1	336	0.378	0.000
	TF101066	Q8UWJ8	LIG_CYCLIN_1	445	0.354	0.000
	TF105165	Q27974	LIG_Clathr_ClatBox_1	496	0.537	0.001
	TF330534	P50895	TRG_LysEnd_APsAcLL_1	604	0.531	0.002
	TF101081	O14757	MOD_PIKK_1	342	0.334	0.007
	TF106101	P04637	TRG_NES_CRM1_1	339	0.367	0.014
	TF106101	P04637	MOD_SUMO	385	0.335	0.020
	TF106101	P04637	MOD_PIKK_1	12	0.412	0.048
	TF315430	P22736	MOD_PKB_1	346	0.243	0.051
	TF332149	O75074	TRG_LysEnd_GGAAcLL_1	764	0.291	0.069
	TF105566	Q01094	LIG_CYCLIN_1	90	0.204	0.077
	TF101081	O14757	MOD_PIKK_1	314	0.303	0.106
	TF106101	P04637	LIG_MDM2	19	0.262	0.126
	TF300772	P49736	MOD_PIKK_1	105	0.350	0.248
	TF106510	P06536	MOD_PIKK_1	524	0.171	0.329
	TF314605	O00203	LIG_Clathr_ClatBox_1	818	0.165	0.558
	TF313542	P49418	LIG_Clathr_ClatBox_2	381	0.255	0.627
	TF101002	P18606	LIG_APCC_Dbox_1	40	0.158	0.666
	TF105842	Q03834	LIG_PCNA	27	0.146	0.706
	TF101056	P30307	LIG_APCC_KENbox_2	150	0.217	0.759
	TF315028	P12887	LIG_PCNA	21	0.158	0.866
	TF101087	O14731	LIG_CYCLIN_1	486	0.214	0.866
	TF318574	Q9UJY5	TRG_LysEnd_GGAAcLL_2	355	0.086	0.888
MIXED GLOB	TF332780	P04004	LIG_RGD	64	0.644	0.000
	TF318283	P46061	MOD_SUMO	525	0.623	0.000
	TF300901	P23396	LIG_MAPK_2	77	0.389	0.000
	TF330851	P10912	LIG_SH2_STAT5	566	0.373	0.002
	TF333209	P54274	MOD_PIKK_1	216	0.592	0.003
	TF330851	P10912	LIG_SH2_STAT5	534	0.303	0.027
	TF101201	P18887	MOD_PIKK_1	368	0.500	0.168
	TF105722	P35251	LIG_RB	662	0.314	0.365
	TF330851	P10912	LIG_SH2_STAT5	627	0.151	0.644

Kolmogorov-Smirnov test comparing the IUP_{diff} distribution of the P_{LM} and A_{LM} sets of each instance. The difference is the Kolmogorov-Smirnov statistic calculated from the cumulative distributions of the compared samples.

[a] protein and module structural classes
