# Supplementary material for: Evidence for the Concerted Evolution between Short Linear Protein Motifs and Their Flanking Regions
Source: PLoS One. 2009 Jul 8;4(7):e6052. doi: 10.1371/journal.pone.0006052 (PMC2702822; doi:10.1371/journal.pone.0006052)
Supplement: Table S2 — Mean and standard deviation of the correlation between PLM and ALM frequency profiles. Spearman correlation coefficient calculated between the PLM and ALM frequency profiles of each instance. Correlation of the frequency profiles of IUPdiff versus locCons and IUPdiff versus globCons are indicated as locCons corr and globCons corr respectively. (0.02 MB PDF) [file pone.0006052.s003.pdf]

Table S2. Mean and standard deviation of the correlation between P_{LM} and A_{LM} frequency profiles

protein class	module class	number[a]	*locCons* corr		*globCons* corr	
			mean	stdev	mean	stdev
DIS	DIS	17	0.20	0.22	0.22	0.17
	GLOB	4	0.34	0.28	0.22	0.20
GLOB	DIS	10	0.34	0.20	0.21	0.18
	GLOB	7	0.23	0.24	0.12	0.22
MIXED	DIS	9	0.11	0.14	0.02	0.10
	GLOB	6	0.12	0.19	0.10	0.20

Spearman correlation coefficient calculated between the P_{LM} and A_{LM} frequency profiles of each instance. Correlation of the frequency profiles of IUP_{diff} versus $locCons$ and IUP_{diff} versus $globCons$ are indicated as $locCons$ corr and $globCons$ corr respectively.

[a] number of instances per structural class
