# Supplementary material for: Evidence for the Concerted Evolution between Short Linear Protein Motifs and Their Flanking Regions
Source: PLoS One. 2009 Jul 8;4(7):e6052. doi: 10.1371/journal.pone.0006052 (PMC2702822; doi:10.1371/journal.pone.0006052)
Supplement: Table S3 — Effect of the stringency of the regular expression matching on the correlation between the PLM and ALM frequency profiles. Spearman correlation coefficient calculated between the PLM and ALM frequency profiles of each instance. Correlation of the frequency profiles of IUPdiff versus locCons and IUPdiff versus globCons are indicated as locCons corr and globCons corr respectively. Percentages indicate the stringency used to define a match to the ELM regular expression: 100% stringency supposes that a LM is present only if there is a perfect match to the ELM regular expression in the same position of the annotated instance; lower percentages consider that a LM is present also in case of partial match to the regular expression. Correlation values in bold show the biggest difference (more than 0.05) with the corresponding 100% stringency correlation value. Missing values can not be calculated due insufficient number of sequence pairs in the ALM set. (0.05 MB PDF) [file pone.0006052.s004.pdf]

Table S3. Effect of the stringency of the regular expression matching on the correlation between the P_{LM} and A_{LM} frequency profiles

structural class[a]	TreeFam id	UniProt id	ELM id	start	*locCons* corr 100%	*locCons* corr 75%	*locCons* corr 25%	*globCons* corr 100%	*globCons* corr 75%	*globCons* corr 25%
	TF106427	P29374	LIG_RB	957	-0.14	-0.14	**0.24**	0.12	0.12	**0.22**
	TF106496	P25054	TRG_NES_CRM1_1	163	-0.09			-0.05		
	TF316358	P10636	LIG_SH3_1	565	-0.05	-0.05		0.42	0.42	
	TF300785	P51531	LIG_RB	1294	-0.01	-0.01	**0.05**	0.12	0.12	**0.23**
	TF314303	O15147	LIG_SH3_5	389	-0.01	**-0.10**	**0.12**	0.16	**0.04**	0.11
	TF325994	P35568	LIG_14-3-3_3	267	0.12	**-0.02**		0.29	**0.12**	
	TF331759	O60315	LIG_CtBP	785	0.16	0.19		0.20	0.24	
	TF323952	P17535	LIG_COP1	241	0.16	**0.32**		0.33	**0.14**	
DIS DIS	TF325994	P35568	LIG_14-3-3_3	371	0.29	0.25	**0.44**	0.42	**0.31**	**0.19**
	TF318445	O35973	TRG_NES_CRM1_1	488	0.32	0.33	0.33	0.50	0.50	0.50
	TF325994	P35570	LIG_SH2_GRB2	896	0.45	0.45	**0.11**	0.52	0.52	**0.22**
	TF101166	P05205	LIG_RB	61	0.53	0.53		0.10	0.10	
	TF320471	P35712	LIG_CtBP	424	0.36	0.36	**0.01**	0.15	0.14	**0.02**
	TF313876	Q91VZ6	LIG_Clathr_ClatBox_1	192	0.31	0.33	0.35	0.16	**0.26**	**0.09**
	TF325994	P35570	LIG_SH2_PTP2	1179	0.23			0.21		
	TF331759	O60315	LIG_CtBP	859	0.34			0.31		
	TF323952	P05412	MOD_PIKK_1	246	0.55	0.55		0.52	0.52	
	TF105306	Q00987	MOD_PIKK_1	392	-0.02	-0.02	**0.07**	0.02	0.02	**0.07**
DIS GLOB	TF323952	P05412	LIG_MAPK_1	32	0.55	0.58	0.58	0.27	0.29	0.29
	TF314861	Q05140	LIG_PIP2_ANTH_1	28	0.51			0.36		
	TF325994	P35570	MOD_CK2_1	96	0.48	0.48	**0.14**	0.39	0.39	**0.27**
	TF335892	P04235	TRG_LysEnd_APsAcLL_1	138	0.18	0.18	**-0.06**	0.28	0.28	**-0.07**
	TF300460	Q04656	TRG_LysEnd_APsAcLL_1	1483	0.38	0.38		0.00	0.00	
	TF105137	Q02750	LIG_MAPK_1	3	0.34	0.39	0.39	0.11	0.10	0.10
	TF300618	P27797	TRG_ER_KDEL_1	414	0.53	0.53		0.22	0.22	
GLOB DIS	TF105135	P45985	LIG_MAPK_1	40	0.36	**0.46**	**0.46**	0.28	**0.34**	**0.34**
	TF105115	Q99683	LIG_14-3-3_1	963	0.33	**0.27**	**0.11**	0.31	**0.25**	**0.14**
	TF300540	P04040	TRG_PTS1	523	0.38	**0.44**	**0.56**	0.36	0.38	**0.29**
	TF105044	P36604	TRG_ER_KDEL_1	660	0.45	**-0.10**	**-0.10**	0.42	**-0.08**	**-0.08**
	TF106381	P09103	TRG_ER_KDEL_1	506	0.52	0.50		0.48	**0.33**	
	TF105042	P17156	LIG_TPR	630	0.65	**0.47**	**0.37**	0.52	**0.65**	**0.60**
	TF335892	P19377	MOD_TYR_ITAM	146	-0.05	-0.05	-0.05	-0.08	-0.08	-0.08
	TF101211	Q8AY27	MOD_PIKK_1	2	0.03	0.03		-0.00	-0.00	
	TF101004	P24385	LIG_RB	5	0.22	0.19	0.19	0.10	**0.04**	**0.04**
GLOB GLOB	TF105115	Q99683	LIG_RB	916	0.19	0.19		0.17	0.17	
	TF105122	P28562	LIG_MAPK_2	339	0.44	0.44	**0.13**	0.25	0.20	**0.01**
	TF315491	P27918	MOD_CMANNOS	318	0.37	**0.43**		0.27	0.31	
	TF105331	Q96GD4	LIG_APCC_Dbox_1	314	0.65	0.65	**0.46**	0.53	0.53	**0.44**
	TF316520	O00268	LIG_HP1_1	762	-0.07			0.01		
	TF101065	Q12834	LIG_APCC_KENbox_2	96	-0.01	-0.01		0.04	0.04	
	TF313542	P49418	LIG_AP2alpha_1	324	0.03	0.03	**-0.09**	0.15	0.15	**0.08**
	TF300772	P49736	MOD_PIKK_1	105	0.09	0.09	0.05	0.16	0.16	0.11
MIXED DIS	TF105351	P35465	LIG_SH3_2	13	0.11	0.11	**-0.09**	-0.06	-0.09	-0.08
	TF332149	O75074	TRG_LysEnd_GGAAcLL_1	764	0.35	**0.27**	**0.27**	0.01	-0.02	-0.02
	TF106101	P04637	TRG_NES_CRM1_1	339	0.04	0.04	0.04	0.01	-0.01	-0.01
	TF318574	Q9UJY5	TRG_LysEnd_GGAAcLL_2	355	0.34	0.34		0.03	0.02	
	TF101089	P53350	LIG_APCC_Dbox_1	336	0.33	0.30	0.38	0.14	0.11	**0.39**
	TF105722	P35251	LIG_RB	662	-0.11	-0.11		-0.06	-0.06	
	TF300901	P23396	LIG_MAPK_2	77	0.24	0.24		0.41	0.41	
MIXED GLOB	TF333209	P54274	MOD_PIKK_1	216	-0.08	-0.08	**0.04**	-0.10	-0.10	-0.11
	TF318283	P46061	MOD_SUMO	525	0.10	0.10		-0.07	-0.07	
	TF101066	Q8UWJ8	LIG_CYCLIN_1	445	0.31	0.31	0.31	0.19	0.19	0.19
	TF330851	P10912	LIG_SH2_STAT5	566	0.30	0.24		0.21	0.23	

Spearman correlation coefficient calculated between the P_{LM} and A_{LM} frequency profiles of each instance. Correlation of the frequency profiles of IUP_{diff} versus *locCons* and IUP_{diff} versus *globCons* are indicated as *locCons* corr and *globCons* corr respectively. Percentages indicate the stringency used to define a match to the ELM regular expression: 100% stringency supposes that a LM is present only if there is a perfect match to the ELM regular expression in the same position of the annotated instance; lower percentages consider that a LM is present also in case of partial match to the regular expression. Correlation values in bold show the biggest difference (more than 0.05) with the corresponding 100% stringency correlation value. Missing values can not be calculated due insufficient number of sequence pairs in the A_{LM} set.

[a] protein and module structural classes

As stated in the Methods, the assignation of the sequence pairs to the P_{LM} and A_{LM} sets depends on the adequacy of the ELM regular expression and might overestimate the size of the A_{LM} set. To check the effect of such definition in the correlation calculation, the P_{LM} and A_{LM} sets were constructed requiring different degree of similarity to the regular expression: 100%, 75% and 25%. This variable stringency in the regular expression matching influences the size of the P_{LM} and A_{LM} , augmenting the number of pairs in the P_{LM} set as the required similarity to the regular expression diminishes. Similarity to the regular expression was calculated taking into account the differential information content of variable and rigid positions in the LM pattern, as defined in [1]. Therefore, 75% or 25% stringency in the regular expression matching means that subsequences that conserve 75% or 25% of the information content of the ELM pattern are considered as LM instances.

The average difference between the 100% stringency correlation and 75% stringency correlation is 0.05 both for *locCons* and *globCons* correlations; no relevant changes with reference to the general trends described in the Results are observed. As expected, for the 25% stringency in the regular expression matching such difference increases (0.16 for *locCons* corr and 0.11 for *globCons* corr). Indeed, many of the additional partial matches included in the P_{LM} set can correspond to false positives, since only 25% sequence similarity to the regular expression is required.

The *locCons* and *globCons* correlations seem to be differentially affected by this extreme relaxation in the LM presence definition (i.e. 25% stringency in regular expression matching). For 67% of the instances the 25% stringency *globcons* correlation values are lower than the corresponding 100% stringency values, indicating a bigger dissimilarity between P_{LM} and A_{LM} sets. Instead, for the *locCons* correlation this is the case only for 47% of the instances. This could suggest a greater stability of the *locCons* as a sequence conservation metric, when studying the evolutionary behaviour of regions flanking LMs. Nevertheless further study on boarder dataset is needed to confirm this hypothesis.

References

[1] Chica C, Labarga A, Gould C, López R, Gibson T (2008) A tree-based conservation scoring method for short linear motifs in multiple alignments of protein sequences. BMC Bioinformatics 9: 229.
